# Association of inflammatory burden index with prognosis as well as stroke-associated pneumonia in non-surgical patients with spontaneous intracerebral hemorrhage

**DOI:** 10.3389/fneur.2025.1596731

**Published:** 2025-07-09

**Authors:** Xinyu Wang, Lingqian He, QiQi Cui, Jiapeng Zhao, Juexian Song

**Affiliations:** Department of Neurology, Xuanwu Hospital, Capital Medical University, Beijing, China

**Keywords:** intracerebal hemorrhage, prognosis predict, stroke associated pneumonia, biomaker, inflammatory index, NLR

## Abstract

**Aim:**

To assessment the new biomarker, the inflammatory burden index (IBI) and their correlations with clinical outcome as well as stroke-associated pneumonia (SAP) of ICH patients.

**Methods:**

Patients diagnosed as spontaneous intracranial hemorrhage (SICH) were retrospectively screened from December 2018 to December 2022. The IBI was formulated as C-reactive protein×neutrophils/lymphocytes. In addition, systemic-inflammatory indices including ratios of neutrophil-to-lymphocyte (NLR), monocyte-to-lymphocyte (MLR), platelet-to-lymphocyte (PLR),and patelet-to-neutrophil (PNR), systemic immune-inflammation index (SII), and systemic inflammation response index (SIRI) on admission also were calculated. The primary outcome measure was 90-day functional outcome and the occurrence of SAP, with an mRS 3–6 score representing a unfavorable outcome. We used ROC curves to evaluate the diagnostic value of these differences. Univariate and multivariable analyses was conducted to identify the factors independently associated with 3 months unfavorable functional outcomes and stroke-associated pneumonia.

**Results:**

A total of 209 patients were enrolled, of which 144 (68.9%) had unfavorable 90-day functional outcome, and 89 (42.1%) ICH patients developed SAP. Elevated IBI is an independent predictor for both stroke-associated pneumonia (SAP) and unfavorable 3-month outcomes in acute ICH patients, and showed a good predictive ability for SAP (AUC = 0.811, *p* < 0.001) and poor prognosis at 3 months (AUC = 0.745, *p* < 0.001).

**Conclusion:**

Inflammatory biomarker index (IBI) are predictive of 90-day functional outcome and the SAP occurrence in patients with ICH.

## Introduction

Intracerebral hemorrhage (ICH) is a particularly devastating form of stroke. Although it represents merely 10–20% of all stroke cases, it accounts for approximately 44% of stroke-related mortality and 60–80% of survivors experience serious neurological impairment, underscoring its disproportionately high clinical impact (1, 2). Most survivors of ICH continue to face complications, stroke-associated pneumonia (SAP) is the most common devastating complication, with an incidence rate of 34.6%, which also have a significant impact on the patient’s overall prognosis (3). Therefore, it is essential to find an early screening strategy for diagnosis and prognosis prediction in ICH patients (4–6). The inflammatory response contributes to the ICH-induced secondary brain injury and is indispensable in the occurrence and progression of SAP (7).

Within hours to days following ICH, a series of inflammatory reactions around the hematoma contributes to secondary injuries and worsen the prognosis (8). Correspondingly, peripheral blood inflammatory biomarkers, as easily accessible markers, may help predict functional outcomes (9). Previous studies have found that systemic inflammatory indices such as the neutrophil-to-lymphocyte ratio (NLR), platelet-to-lymphocyte ratio (PLR), monocyte-to-lymphocyte ratio (MLR), and systemic inflammatory response index (SIRI) are correlated with the clinical outcomes and complications of intracerebral hemorrhage (ICH) patients (9–15). The Inflammatory Burden Index (IBI) is a novel inflammatory marker defined as C-reactive protein (CRP) multiplied by the neutrophil-to-lymphocyte ratio (NLR) (16–18). Reflecting the level of inflammatory burden in tumors, IBI has been proven to be a strong prognostic predictor for cancer patients and may be the optimal indicator among various systemic inflammation indicators.

Accordingly, in this study, we aimed to investigate the association between IBI and the prognosis as well as in-hospital complications of ICH patients. We also compared the prognostic performance of IBI with previously reported inflammatory biomarkers to determine their prognostic value.

## Methods

### Patient selection

Patients diagnosed as spontaneous intracranial hemorrhage (SICH) at a comprehensive stroke center were retrospectively screened from December 2018 to December 2022. The inclusion criteria were as follows:

(1) Diagnosis of spontaneous SICH, confirmed first (or recurrent) stroke by computerized tomography (CT).
(2) Within 72 h from the onset.
(3) Aged 18 years or older.

The exclusion criteria were:

(1) Secondary intracranial hemorrhage, caused by trauma, brain tumor, ruptured aneurysm, vascular malformation, venous sinus thrombosis, or other known causes.
(2) Pre-stroke disability [a premorbid modified Rankin scale (mRS) score of ≥3].
(3) Patients who required surgical intervention.
(4) Severe hepatic or renal dysfunction.
(5) The presence of systemic inflammatory rheumatic diseases, hematologic diseases, chronic respiratory diseases, systemic or localized infectious diseases, and active cancer; concurrent/recent infectious diseases, or ongoing anti-inflammatory or immunomodulatory therapies.
(6) Missing clinical or follow-up information.

### Clinical, imaging, and outcome data collection

The detailed data collected from the electronic medical record system were as follows:

(1) Demographic data (sex, age, smoker and alcohol drinker).
(2) Medical history (hypertension, diabetes, and previous history of stroke).
(3) Initial imaging (hemotoma volume [calculated by a*b*c/2], the presence of IVH).
(4) Admission status (NIHSS, GCS score, Systolic BP, mmHg, Diastolic BP, mmHg).
(5) Laboratory tests (CRP, neutrophils, lymphocytes, glucose, hemoglobin, leukocytes, platelets and monocytes).

The primary outcome of the study was 90-day unfavorable outcome, defined as a modified Rankin Scale (mRS) score of 3–6. The secondary outcome was stroke-associated pneumonia (SAP). According to the recommendations of the Pneumonia in Stroke Consensus Group, SAP was defined as the spectrum of lower respiratory tract infections within the first 7 days after stroke onset; (19) It was diagnosed on a basis of clinical symptoms (eg, cough, purulent sputum), signs (eg, fever, tachypnea), or laboratory investigations (eg, white blood cell count, C-reactive protein), and supported by typical chest radiographs findings.

All outcomes assessments were assessed by certified neurologists who were blinded to the clinical data.

### Inflammatory biomarkers

Blood samples were routinely collected for the blood counts (including neutrophils, monocytes, and lymphocytes) assessment within 24 h after admission. In cases where multiple laboratory data were obtained during these time period, the highest value was recorded.

Inflammatory Burden Index (IBI) = CRP × neutrophils/lymphocytes,
Neutrophil-to-lymphocyte ratio (NLR) = neutrophils/lymphocytes,
Monocyte-to-lymphocyte ratio (MLR) = monocytes/lymphocytes,
Platelets-to-Lymphocytes Ratio (PLR) = platelets/lymphocytes,
and Systemic Inflammation Response Index (SIRI) = neutrophils × monocytes/lymphocytes.

### Statistical analysis

The continuous variables were expressed as the mean (standard deviation; SD) or median (interquartile range; IQR), and the categorical variables were presented as counts (percentages). For continuous variables, intergroup differences were analyzed using Student t-test or the Mann–Whitney *U*-test, as appropriate. We used the Shapiro–Wilk normality test and Q-Q plots to evaluate the normality of the data for comparison of categorical variables, the chi-squared test was used. Multivariable logistic regression analysis was performed to explore the effect of IBI, NLR, PLR, MLR and SIRI on 3-month functional outcomes, while adjusting for potential confounders. We plotted the receiver operating characteristic (ROC) curves to assess the predictive performance of the model. The predictive accuracy of the index was then determined by measuring the specificity, sensitivity and area under the receiver operating characteristic (ROC) curve.

SPSS 22.0 and R 3.6.2 software were employed for statistical analysis with p<0.05 defined as statistical significance.

### Ethical approval

This study was approved by the Ethics Committee of the Xuanwu Hospital, Capital Medical University. It was performed according to the Principles of Declaration of Helsinki. The requirement for informed consent was waived because of its retrospective nature and minimal risk to patients. This study was reported in accordance with Strengthening the Reporting of Observational studies in Epidemiology (STROBE) guidelines (20).

## Results

### Study population and baseline characteristic

A total of 209 patients were included, the median age was 60.0 (53.0–67.0) years, and 147 (70.3%) patients were male. The median NIHSS and GCS scores on admission were 6 (IQR, 3–10) and 15 (IQR, 13–15), respectively. Among the total patients patients, 144 (65.45%) had an unfavorable outcome at 3 months (Table 1).

**Table 1:** Baseline characteristics of the 209 patients with ICH.

Characteristic	mRS Score at 3 Months		
	0–2 (*n* = 65)	3–6 (*n* = 144)	*p* value
Age (median [IQR])	58.00 [47.00, 66.00]	62.50 [54.00, 71.00]	**0.017**
Male, *n* (%)	42 (64.6)	105 (72.9)	0.293
Smoker, *n* (%)	21 (32.8)	55 (39.0)	0.487
Alcohol drinker, *n* (%)	21 (32.8)	56 (39.7)	0.429
Hypertension, *n* (%)	52 (80.0)	125 (86.8)	0.290
Diabetes mellitus, *n* (%)	8 (12.3)	34 (23.6)	0.089
NIHSS, median [IQR]	2.00 [1.00, 4.00]	9.00 [5.00, 12.50]	**<0.001**
GCS score, median [IQR]	15.00 [15.00, 15.00]	15.00 [12.00, 15.00]	**0.012**
SBP, mmHg, mean (SD)	154.50 (26.11)	156.72 (22.65)	0.536
DBP, median [IQR]	90.50 [80.75, 98.25]	90.00 [80.00, 100.00]	0.518
Hematoma_volume, median [IQR]	5.77 [2.20, 14.00]	11.53 [6.23, 19.90]	**0.001**
Edema degree, mean (SD)	2.52 (0.79)	2.85 (0.72)	0.004
Nasogastric tube, *n* (%)	18 (26.8)	41 (28.4)	0.731
Mechanical ventilation, *n* (%)	7 (10.4)	19 (13.1)	0.660
Neutrophils, mean (SD)	5.46 (2.28)	6.98 (3.38)	**0.001**
Lymphocytes, mean (SD)	1.93 (2.14)	1.30 (0.62)	**0.001**
Monocytes, mean (SD)	0.55 (0.72)	0.54 (0.43)	0.814
PLT, mean (SD)	224.36 (60.03)	216.67 (80.97)	0.509
CRP, median [IQR]	3.00 [2.00, 5.25]	7.60 [3.50, 29.00]	**<0.001**
IBI, median [IQR]	8.68 [3.92, 25.48]	34.90 [10.15, 261.38]	**<0.001**
NLR, median [IQR]	3.07 [1.91, 4.83]	4.96 [3.05, 9.11]	**<0.001**
MLR, median [IQR]	0.25 [0.19, 0.36]	0.38 [0.25, 0.60]	**<0.001**
PLR, median [IQR]	125.75 [103.78, 160.00]	152.41 [109.19, 228.52]	**0.008**
SIRI, median [IQR]	3.07 [1.89, 4.82]	4.77 [2.94, 8.75]	**<0.001**

DBP, diastolic blood pressure; SBP, systolic blood pressure; NIHSS, National Institute of Health stroke scale; GCS, Glasgow Coma Scale; ICH, intracerebral hemorrhage; IBI, inflammatory burden index; NLR, neutrophil to lymphocyte ratio; SIRI, systemic inflammation response index; platelet-to-lymphocyte ratio (PLR); monocyte-to-lymphocyte ratio (MLR); SAP, stroke-associated pneumonia. The bold values are the significant values (*p* < 0.05).

**Figure 1:**
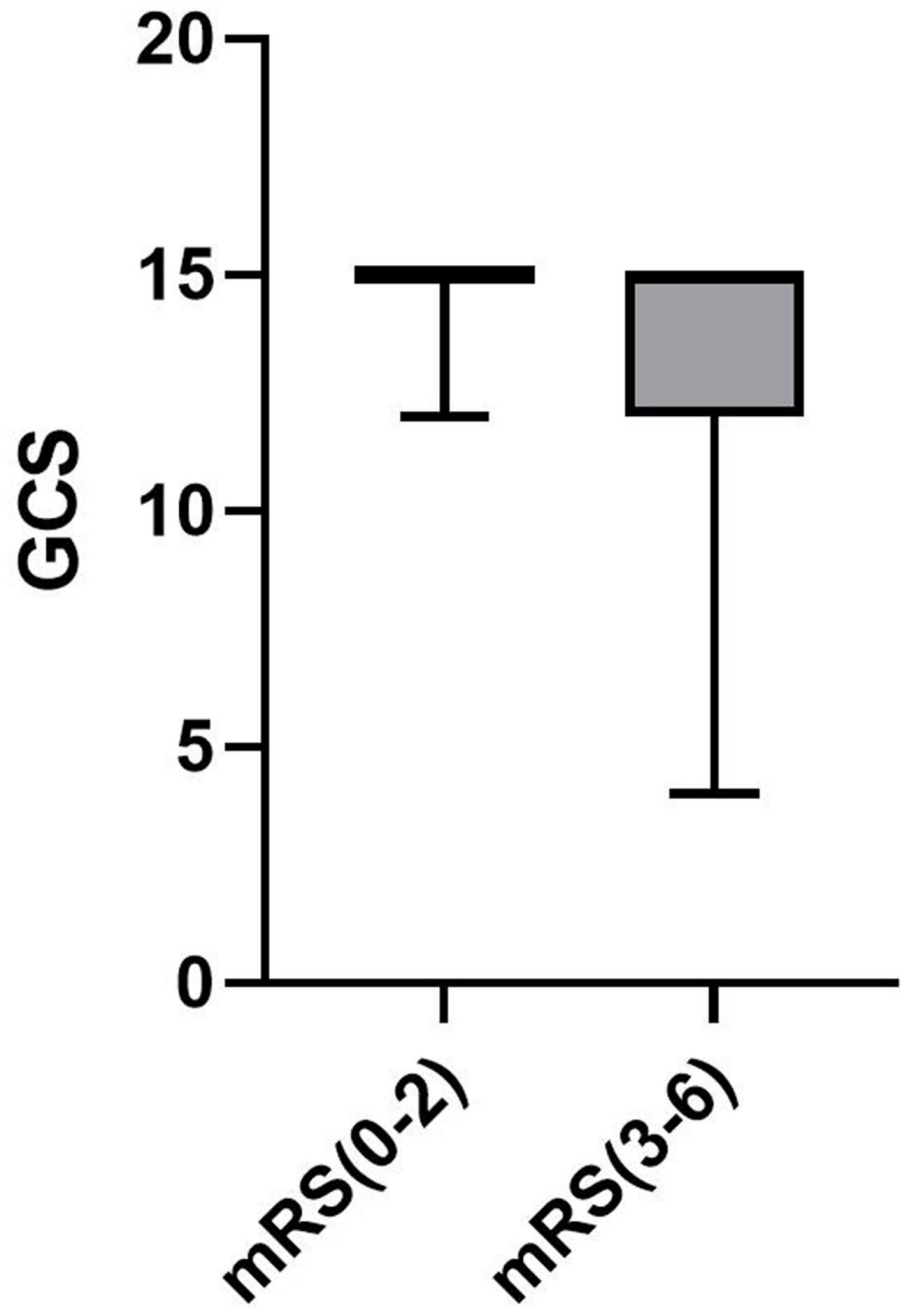
QQ plot of study participant distribution based on Inflammatory indicators.

Patients with unfavorable outcome at 3 months had higher age (*p* = 0.017), lower admission GCS scores (*p* = 0.012), higher NIHSS scores (*p* < 0.001), larger hematoma volumes (*p* < 0.001) and higher edema degree (*p* = 0.004). The distribution of GCS is visualized in Figure 1. Meanwhile, higher IBI (*p* < 0.001), NLR (*p* < 0.001), PLR (*p* = 0.008), SIRI (*p* < 0.001) and CRP (*p* < 0.001) values on admission were observed in patients with unfavorable outcome at 3 months, as shown in Table 1.

### Unfavorable outcome

**Figure 2:**
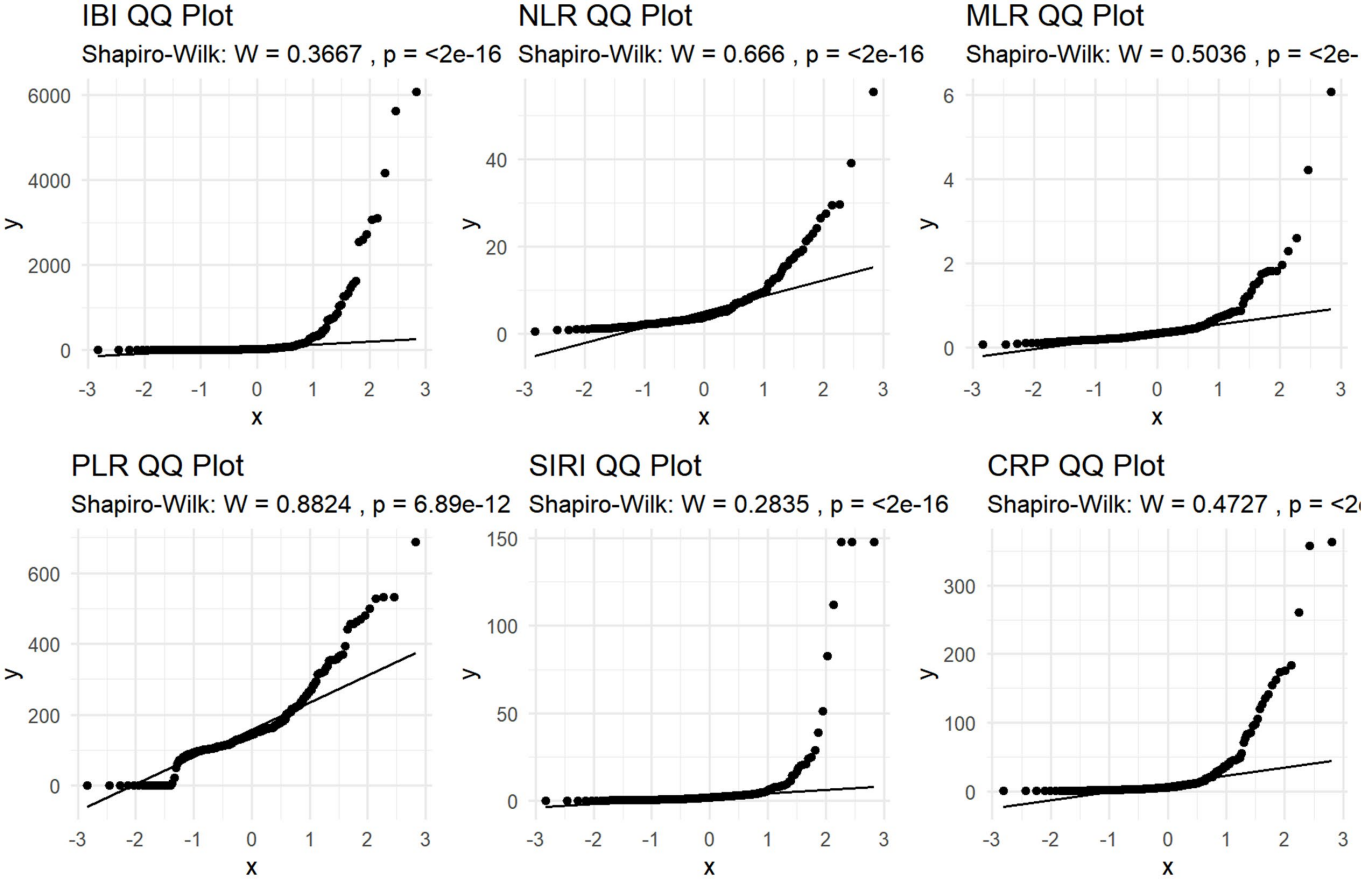
Distribution of GCS.

Shapiro–Wilk normality test and Q-Q plot was performed to evaluate normality assumption. The result showed typical skewness of the data, so logarithmic conversion was performed on it. (Shapiro–Wilk W>0.05; *p* < 0.05; Table 2; Figure 2).

**Table 2:** Skewness, Kurtosis, and Shapiro–Wilk test results for normality assessment of inflammatory indicators.

Variable	Skewness	Kurtosis	Shapiro_W	Shapiro_p
IBI	4.898	30.337	0.367	<0.001
NLR	3.228	17.729	0.666	<0.001
MLR	5.287	40.866	0.504	<0.001
PLR	1.363	5.554	0.882	<0.001
SIRI	5.897	39.134	0.283	<0.001
SII	2.718	14.006	0.734	<0.001

IBI, inflammatory burden index; NLR, neutrophil to lymphocyte ratio; SIRI, systemic inflammation response index; SAP, stroke-associated pneumonia; platelet-to-lymphocyte ratio (PLR); monocyte-to-lymphocyte ratio (MLR).

Univariate analyses showed that the patients with unfavorable outcome had higher levels of lnIBI, lnNLR, lnMLR, lnPLR, lnSIRI and lnCRP; After the adjustment for potential confounders, including age, sex, Diabetes mellitus, NIHSS, Hematoma volume and Edema, multivariate logistic regression analysis revealed that lnIBI (*p* < 0.001, OR = 1.607, 95% CI 1.240–2.144), NLR (*p* = 0.003, OR = 2.232, 95% CI 1.199–4.366), and CRP (*p* = 0.025, OR = 1.518, 95%CI 1.069–2.225) were independent predictors of poor prognosis (Table 3).

**Table 3:** Univariate and multivariate logistics regression analyses for 90 day functional outcome.

90-day mRS	Univariate		Multivariate	
Predictor	OR	*p*_value	OR	*p*_value
Sex	1.444 (0.754–2.764)	0.283	0.771 (0.307–1.884)	0.572
Age	1.037 (1.014–1.061)	**0.004**	1.024 (0.993–1.057)	0.139
Diabetes mellitus	1.922 (0.894–4.133)	0.052	2.361 (0.847–7.096)	0.110
NIHSS	1.246 (1.156–1.344)	**<0.001**	1.459 (1.295–1.681)	**<0.001**
Hematoma volume	1.718 (1.498–2.038)	**0.0379**	0.992 (0.961–1.025)	0.611
Edema	1.765 (1.162–2.680)	**0.004**	1.280 (0.718–2.305)	0.404
ln IBI	1.810 (1.492–2.198)	**<0.001**	1.607 (1.240–2.144)	**<0.001**
ln NLR	2.782 (1.762–4.392)	**<0.001**	2.232 (1.199–4.366)	**0.003**
ln MLR	2.767 (1.621–4.720)	**<0.001**	1.724 (0.899–3.559)	0.118
ln PLR	2.750 (1.438–5.259)	**0.002**	1.505 (0.903–2.783)	0.147
ln SIRI	1.819 (1.308–2.531)	**<0.001**	1.356 (0.893–2.115)	0.161
lnCRP	2.058 (1.514–2.799)	**<0.001**	1.518 (1.069–2.225)	**0.025**

Multivariable regression: adjusted for age, sex, Diabetes mellitus, NIHSS, Hematoma volume and Edema. As indicated by the bold font, the *p* value reached statistical significance.

ln, the natural logarithm; OR, odds ratio; CI, con confidence intervals; IBI, inflammatory burden index; NLR, neutrophil to lymphocyte ratio; SIRI, systemic inflammation response index; SAP, stroke-associated pneumonia; platelet-to-lymphocyte ratio (PLR); monocyte-to-lymphocyte ratio (MLR). The bold values are the significant values (*p* < 0.05).

**Figure 3:**
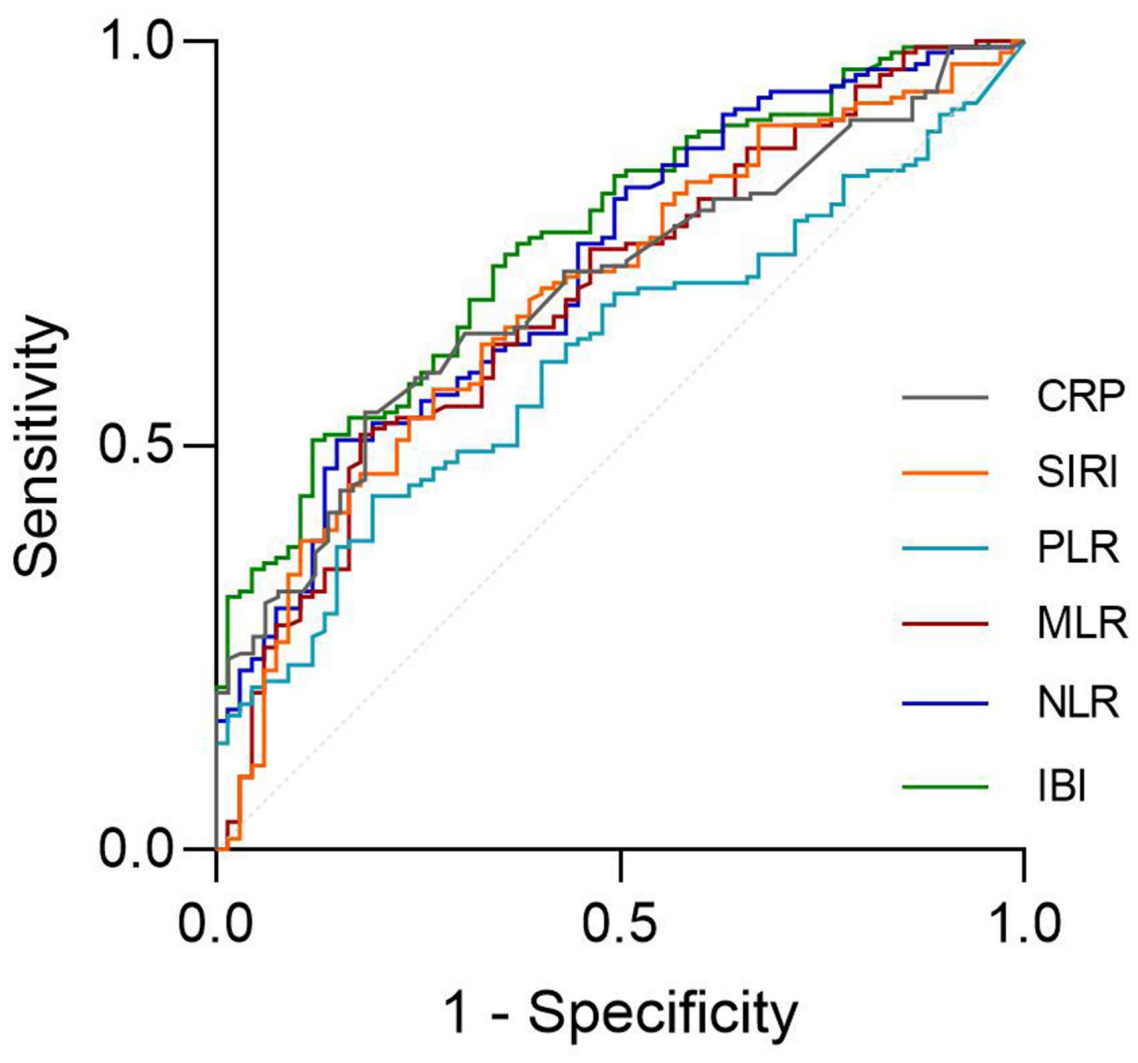
Association between lnIBI and mRS: Restricted Cubic Spline analysis.

**Figure 4:**
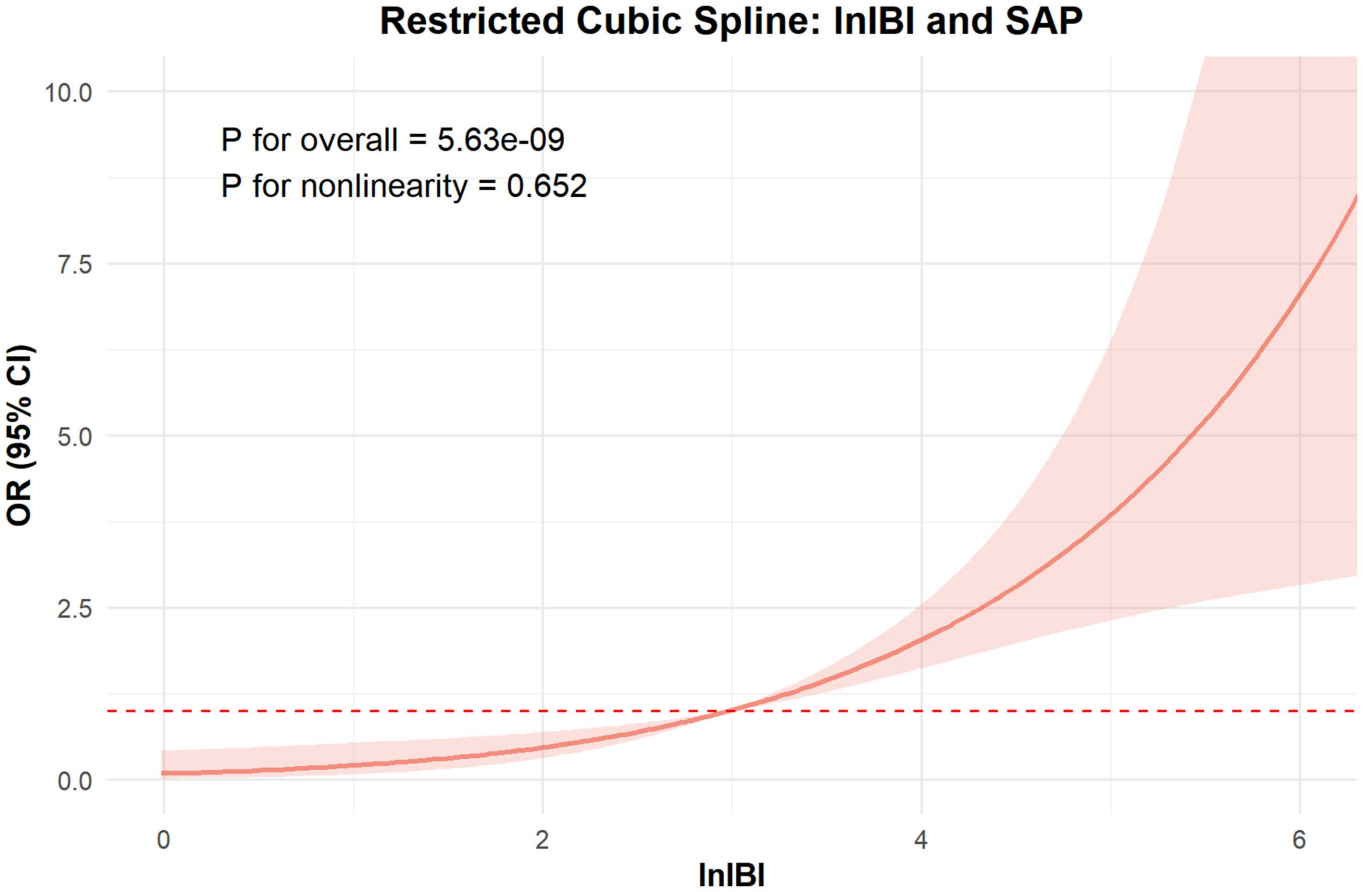
Association between lnIBI and SAP: Restricted Cubic Spline analysis.

The ROC analysis and AUCs with respect to the 90 day outcome are shown in the Figure 3. ROC analysis revealed that IBI had good predictive accuracy for mRS (AUC = 0.745, *p* < 0.001), followed by NLR (AUC = 0.719, *p* < 0.001), without significant difference in AUC between the IBI and NLR (DeLong test, *p* = 0.146). MLR, SIRI and CRP showed moderate predictive value (AUC = 0.673–0.695, all *p* < 0.001), while PLR performed poorly, respectively (AUC = 0.605, *p* = 0.0087) (Figure 4). Details of the optimal cutoff, specificity and sensitivity rates are shown in Table 4.

**Table 4:** AUC in predicting 90 day outcomes.

mRS	AUC	95% CI	Cut off	Sensitivity	Specificity	*p* value
IBI	0.745	0.688 to 0.822	52.356	0.472	0.887	<0.001
NLR	0.719	0.6469 to 0.7916	5.098	0.514	0.806	<0.001
MLR	0.673	0.6087 to 0.7611	0.377	0.514	0.823	<0.001
PLR	0.605	0.5333 to 0.6872	165.899	0.438	0.806	0.00871
SIRI	0.668	0.6090 to 0.7611	2.153	0.569	0.71	<0.001
CRP	0.695	0.6230 to 0.7674	6.995	0.549	0.79	<0.001

AUC, Area Under Curve; CI, con confidence intervals; IBI, inflammatory burden index; NLR, neutrophil to lymphocyte ratio; SIRI, systemic inflammation response index; platelet-to-lymphocyte ratio (PLR); monocyte-to-lymphocyte ratio (MLR).

### SAP occurrence

In univariate analyses, elevated levels of lnIBI, lnNLR, lnMLR, lnPLR, lnSIRI and lnCRP were associated with an increased risk of SAP. In the multivariable logistic regression analysis, after adjusting for confounders factors, including age, sex, Diabetes mellitus, NIHSS, Hematoma volume, Edema, Nasogastric feeding and mechanical ventilation, elevated ln-transformed inflammatory biomarkers remained independent predictors of poor outcomes. NLR (OR = 4.993, 95% CI 2.724–9.919), IBI (OR = 1.813, 95% CI 1.446–2.333) showed particularly strong associations (all *p* < 0.001, Table 5).

**Table 5:** Univariate and multivariate logistics regression analyses for SAP occurrence.

SAP	Univariate		Multivariate	
Predictor	OR	*p*_value	OR	*p*_value
Sex	1.384 (0.761–2.556)	0.291	0.865 (0.385–1.958)	0.724
Age	1.044 (1.022–1.067)	0.001	1.033 (1.004–1.064)	**0.0277**
Diabetes mellitus	1.215 (0.611–2.395)	0.573	0.967 (0.385–2.366)	0.942
NIHSS	1.156 (1.098–1.225)	**<0.001**	1.124 (1.052–1.207)	**0.009**
Hematoma volume	1.036 (1.016–1.060)	0.001	1.017 (0.990–1.047)	0.227
Edema	1.492 (1.032–2.189)	0.036	0.909 (0.528–1.549)	0.727
Nasal	0.888 (0.481–1.619)	0.701	0.796 (0.301–2.008)	0.634
respirator	0.870 (0.363–1.993)	0.7461	0.613 (0.148–2.412)	0.489
ln IBI	2.107 (1.729–2.638)	**<0.001**	1.813 (1.446–2.333)	**<0.001**
ln NLR	6.956 (4.115–12.659)	**<0.001**	4.993 (2.724–9.919)	**<0.001**
ln MLR	3.544 (2.211–5.998)	**<0.001**	2.243 (1.311–4.037)	**0.005**
ln PLR	5.313 (2.749–10.960)	**<0.001**	3.628 (1.742–8.275)	**0.001**
ln SIRI	1.933 (1.472–2.618)	**<0.001**	1.461 (1.080–2.010)	**0.015**
lnCRP	2.156 (1.680–2.849)	**<0.001**	1.661 (1.237–2.281)	**0.001**

Multivariable regression: adjusted for age, sex, Diabetes mellitus, NIHSS, Hematoma volume and Edema. As indicated by the bold font, the p Value reached statistical significance.

ln, the natural logarithm; OR, odds ratio; CI, con confidence intervals; IBI, inflammatory burden index; NLR, neutrophil to lymphocyte ratio; SIRI, systemic inflammation response index; platelet-to-lymphocyte ratio (PLR); monocyte-to-lymphocyte ratio (MLR). The bold values are the significant values (*p* < 0.05).

**Figure 5:**
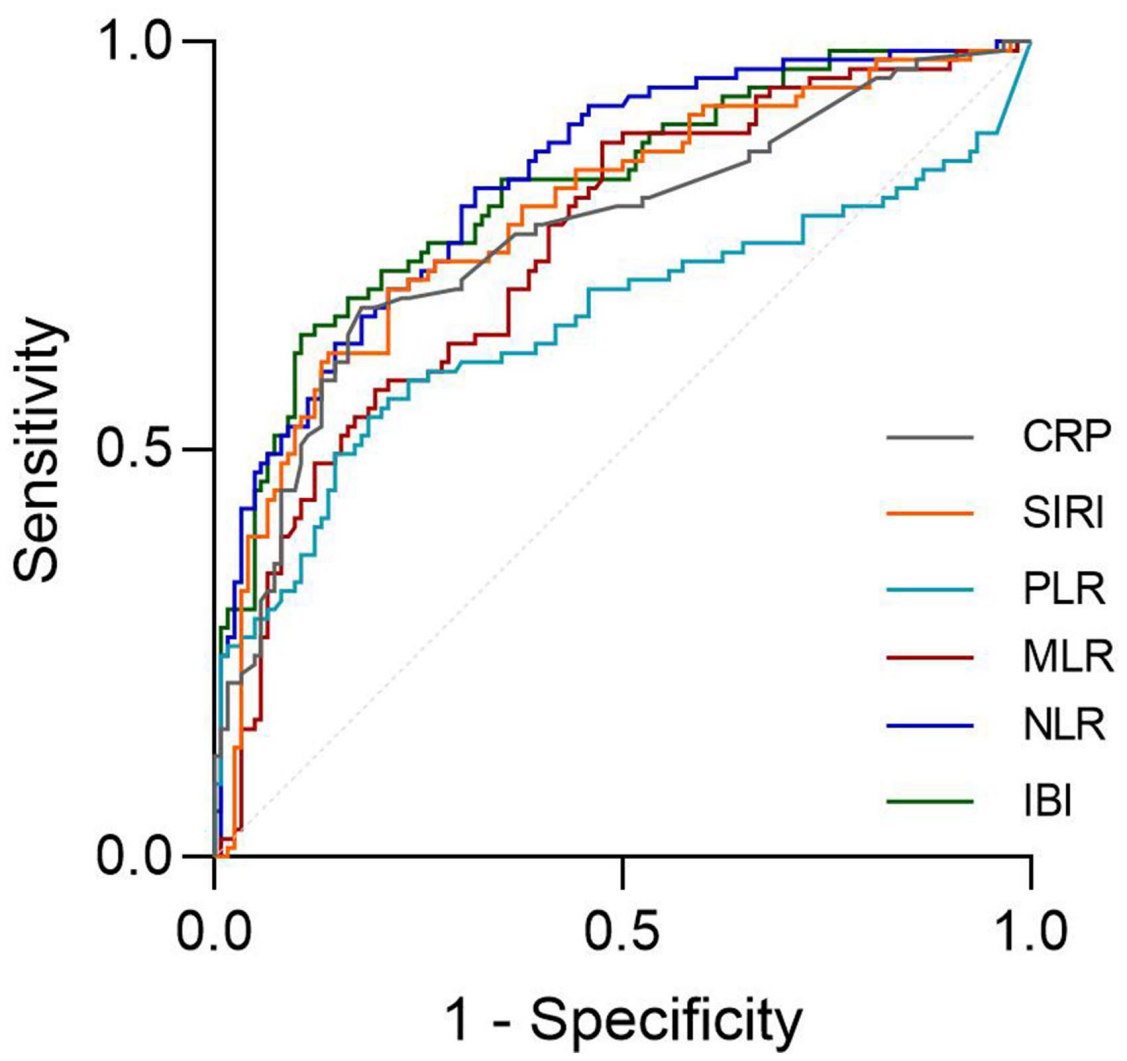
The ROC value of CRP, SIRI, PLR, MLR, NLR and IBI in predicting 3-months poor functional outcomes in ICH patients.

The ROC curves demonstrated that NLR and IBI presented a high predictive value for SAP occurence (Figure 5, AUC = 0.811, 0.814, respectively), without significant difference in AUC between the IBI and NLR (DeLong test, *p* = 0.881). Details of the optimal cutoff, specificity and sensitivity rates are shown in Table 6.

**Table 6:** AUC in predicting SAP occurrence.

SAP	AUC	95%CI	Cut off	Sensitivity	Specificity	*p* value
IBI	0.811	0.751–0.871	62.985	0.64	0.889	<0.001
NLR	0.814	0.756–0.872	3.666	0.82	0.641	<0.001
MLR	0.741	0.673–0.809	0.279	0.876	0.513	<0.001
PLR	0.652	0.571–0.734	173.689	0.539	0.812	<0.001
SIRI	0.781	0.716–0.846	2.433	0.697	0.778	<0.001
CRP	0.757	0.689–0.825	6.995	0.674	0.795	<0.001

AUC, Area Under Curve; CI, con confidence intervals; IBI, inflammatory burden index; NLR, neutrophil to lymphocyte ratio; SIRI, systemic inflammation response index; platelet-to-lymphocyte ratio (PLR); monocyte-to-lymphocyte ratio (MLR).

**Figure 6:**
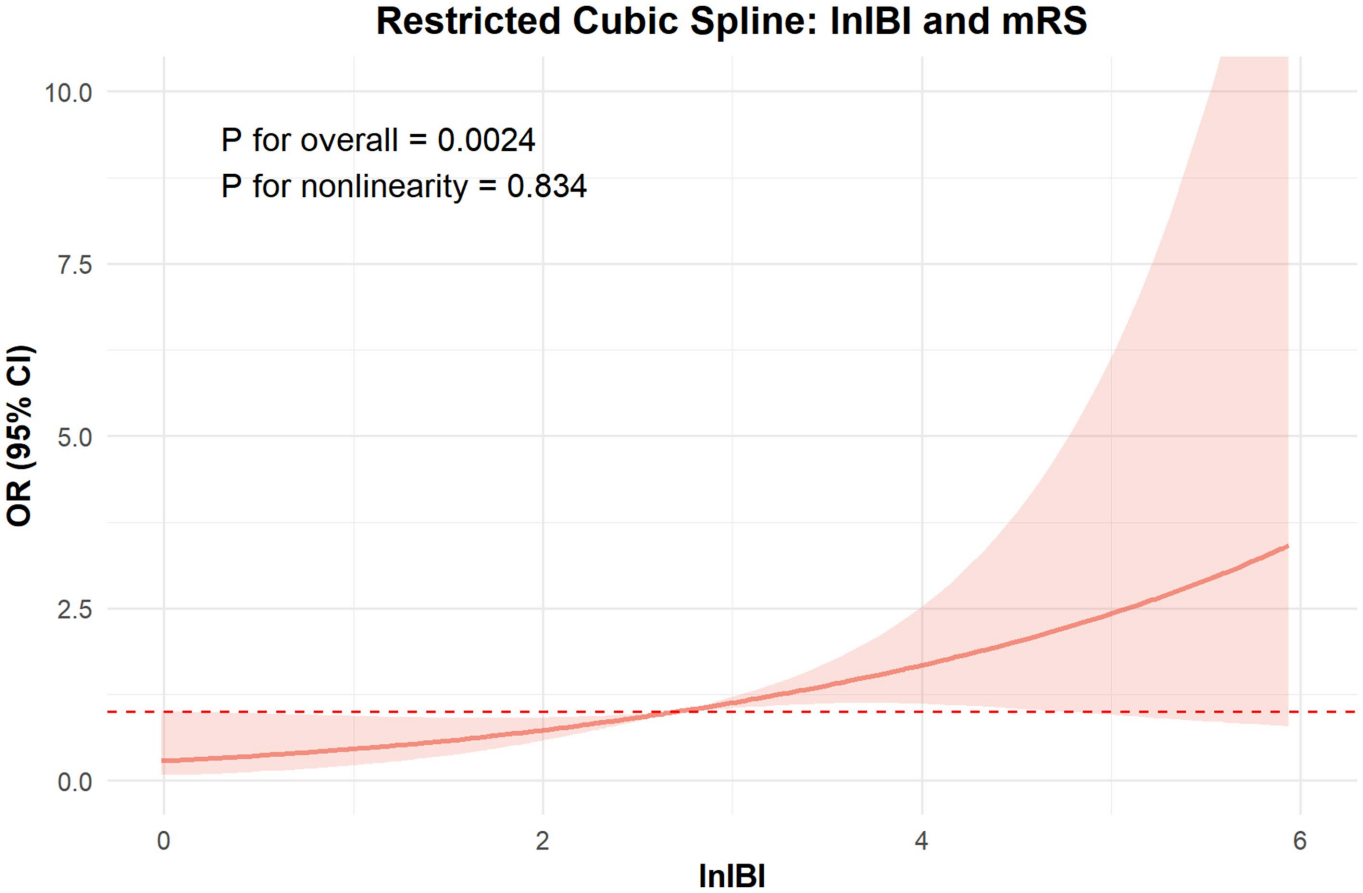
The ROC value of CRP, SIRI, PLR, MLR, NLR and IBI in predicting SAP in ICH patients.

The RCS analysis revealed no nonlinear relationship between IBI and the occcurance of SAP and 90 day function outcome (*p* for overall < 0.01, *p* for nonlinear = 0.652, 0.834, respectively). The risk of SAP and poor prognosis was progressively increased with increasing inflammatory markers, without a clear inflection point (Figure 6).

### Discussion

The main findings of our study are that elevated IBI are independent risk factors for SAP and showed a good predictive ability for SAP (AUC = 0.811, *p* < 0.001). The elevated IBI is independent predictor of unfavorable outcomes at 3 months in patients with acute ICH and showed moderate predictive ability for poor prognosis at 3 months (AUC = 0.745, *p* < 0.001). Furthermore, in comparisons of various inflammatory indicators, we observed that IBI consistently showed significant and stable results in assessing stroke prognosis and SAP.

Systemic inflammation plays a significant role in the secondary injury of ICH. Accumulating evidences indicate readily available serum biomarkers of inflammation can be efficient predictive indicators of prognosis in patients with ICH (21–23). Similar to the findings of us, Wang observed that among the NLR, SII, PLR, SIRI, the NLR was the best predictor for SAP occurrence and a poor outcome at 3 months in ICH patients. However, the limitations of their study are that their study only shows the predictive power of the SII and SIRI for mRS scores at 3 months (13). A meta-analysis of 14 studies showed that NLR independently predicted 90-day prognosis, but due to substantial heterogeneity among the included studies, the results should be view with caution (11). Therefore, a quest for more reliable predictive inflammatory markers of the prognosis and complications of ICH patients is still warranted.

The inflammatory biomarker index (IBI) has been identified as a more reliable prognostic marker for cancer compared to other combinations of inflammatory biomarkers. One study reported that elevated IBI levels at admission were associated with poor functional outcomes and complications in patients with subarachnoid hemorrhage (SAH) (24). Another study demonstrated that higher IBI levels were an independent predictor of poor outcomes in ischemic stroke patients undergoing endovascular therapy (EVT) (16). However, to date, no studies have explored the relationship between IBI and the prognosis or incidence of complication in patients with cerebral hemorrhage.

Our findings demonstrate that IBI, NLR, and CRP are independent predictors of poor prognosis and SAP (*p* < 0.05), in which IBI has a relatively more robust association with the prognosis at 3 months and complications of aSAH patients. A potential explanation for this advantage is that NLR integrate information from neutrophils and Lymphocytes, reflecting the balance between innate (neutrophils) and adaptive (lymphocytes) immune responses. The impact of NLR on neurological outcome and SAP has been demonstrated by previous studies (13, 25–27). C-reactive protein (CRP) is acute-phase proteins induced by inflammatory factors, plasma CRP have been proven to be an independent predictor of ICH outcome, early hematoma growth and early neurological worsening (28, 29). In combination, IBI may be more valuable and reliable biomarkers for predicting outcomes across various diseases, providing both acute and immune inflammatory information (17).

Neutrophils is the first peripheral blood cell type to infiltrate perihematomal tissue and the hematoma itself following ICH. Neutrophils promote the release of free radicals and matrix metalloproteinase-9, which lead to cellular injury, endothelial basement membrane injury and the destruction of brain–blood barrierr and the increase of tissue edema around cerebral hematoma (8, 30). It was found that autoreactive T cells could promote vascular reconstruction and healing after cerebral trauma. Hence, the increase of blood neutrophils would increase Blood–Brain Barrier breakdown, while increase of blood regulatory T lymphocytes could alleviate the degradation of Blood–Brain Barrier (31, 32). Research indicates that elevated CRP can lead to blood–brain barrier (BBB) disruption through CD16/CD32-mediated p38-MAPK-dependent ROS formation and activation of MLCK, which triggers the cellular contractile machinery and consequently causes BBB breakdown (33). As a result, elevated IBI (an increase of blood neutrophils or decrease of lymphocytes) might aggravate the degradation of Blood–Brain Barrier and worsen Brain damage.

Our research has several limitations. First, it is a retrospective, single-center study, the number of patients in this cohort is limited. Accordingly, the possibility of Type II error cannot be ruled out. Second, only inflammatory indexes within the first 24 h of admission was collected as an observation index, and the effect of dynamic indicators change on prognosis was not evaluated. Thus, future studies are needed to explore the changes and predictive value of inflammatory factors at different time points after onset. Third, all of the patients included in the present study were Chinese, further studies are needed to investigate if these results are reproducible in other populations.

In our study of 209 patients with ICH, we demonstrated that the inflammatory biomarker index (IBI) has significant clinical value in predicting poor functional outcomes and SAP. IBI reflects both the likelihood of secondary brain injury and the susceptibility to post-stroke complications. Therefore, monitoring IBI can assist clinicians in identifying patients at higher risk of poor prognosis, particularly in emergency settings.

## Data Availability

The original contributions presented in the study are included in the article/supplementary material, further inquiries can be directed to the corresponding author.

## References

[1] GBD 2019 Stroke Collaborators. Global, regional, and national burden of stroke and its risk factors, 1990-2019: a systematic analysis for the global burden of disease study 2019. Lancet Neurol. (2021) 20:795–820. doi: 10.1016/S1474-4422(21)00252-0, PMID: 34487721 PMC8443449

[2] Ma Q Li R Wang L Yin P Wang Y Yan C. Temporal trend and attributable risk factors of stroke burden in China, 1990-2019: an analysis for the global burden of disease study 2019. Lancet Public Health. (2021) 6:e897–906. doi: 10.1016/S2468-2667(21)00228-0 34838196 PMC9047702

[3] Wang YJ Li ZX Gu HQ Zhai Y Zhou Q Jiang Y. China stroke statistics: an update on the 2019 report from the National Center for healthcare quality Management in Neurological Diseases, China National Clinical Research Center for neurological diseases, the Chinese Stroke Association, National Center for chronic and non-communicable disease control and prevention, Chinese Center for Disease Control and Prevention and institute for global neuroscience and stroke collaborations. Stroke Vasc Neurol. (2022) 7:415–50. doi: 10.1136/svn-2021-001374, PMID: 35443985 PMC9614174

[4] Koennecke HC Belz W Berfelde D Endres M Fitzek S Hamilton F. Factors influencing in-hospital mortality and morbidity in patients treated on a stroke unit. Neurology. (2011) 77:965–72. doi: 10.1212/WNL.0b013e31822dc795, PMID: 21865573

[5] Ziai WC. Hematology and inflammatory signaling of intracerebral hemorrhage. Stroke. (2013) 44:S74–8. doi: 10.1161/STROKEAHA.111.000662, PMID: 23709738 PMC12054399

[6] Chu H Huang C Zhou Z Tang Y Dong Q Guo Q. Inflammatory score predicts early hematoma expansion and poor outcomes in patients with intracerebral hemorrhage. Int J Surg. (2023) 109:266–76. doi: 10.1097/JS9.0000000000000191, PMID: 37093070 PMC10389560

[7] Ohashi SN DeLong JH Kozberg MG Mazur-Hart DJ van Veluw SJ Alkayed NJ. Role of inflammatory processes in hemorrhagic stroke. Stroke. (2023) 54:605–19. doi: 10.1161/STROKEAHA.122.037155 36601948

[8] Xue M Yong VW. Neuroinflammation in intracerebral haemorrhage: immunotherapies with potential for translation. Lancet Neurol. (2020) 19:1023–32. doi: 10.1016/S1474-4422(20)30364-1, PMID: 33212054

[9] Shtaya A Bridges LR Williams R Trippier S Zhang L Pereira AC. Innate immune anti-inflammatory response in human spontaneous intracerebral hemorrhage. Stroke. (2021) 52:3613–23. doi: 10.1161/STROKEAHA.121.034673, PMID: 34281379 PMC7611898

[10] Lattanzi S Brigo F Trinka E Cagnetti C di Napoli M Silvestrini M. Neutrophil-to-lymphocyte ratio in acute cerebral hemorrhage: a system review. Transl Stroke Res. (2019) 10:137–45. doi: 10.1007/s12975-018-0649-4, PMID: 30090954

[11] Shi M Li XF Zhang TB Tang QW Peng M Zhao WY. Prognostic role of the neutrophil-to-lymphocyte ratio in intracerebral hemorrhage: a systematic review and Meta-analysis. Front Neurosci. (2022) 16:825859. doi: 10.3389/fnins.2022.825859, PMID: 35360156 PMC8960242

[12] Kim Y Sohn JH Kim C Park SY Lee SH. The clinical value of neutrophil-to-lymphocyte ratio and platelet-to-lymphocyte ratio for predicting hematoma expansion and poor outcomes in patients with acute intracerebral hemorrhage. J Clin Med. (2023) 12:3004. doi: 10.3390/jcm12083004, PMID: 37109337 PMC10145379

[13] Wang RH Wen WX Jiang ZP Du ZP MZH Lu AL. The clinical value of neutrophil-to-lymphocyte ratio (NLR), systemic immune-inflammation index (SII), platelet-to-lymphocyte ratio (PLR) and systemic inflammation response index (SIRI) for predicting the occurrence and severity of pneumonia in patients with intracerebral hemorrhage. Front Immunol. (2023) 14:1115031. doi: 10.3389/fimmu.2023.1115031 36860868 PMC9969881

[14] Li Y Wen D Cui W Chen Y Zhang F Yuan M. The prognostic value of the acute phase systemic immune-inflammation index in patients with intracerebral hemorrhage. Front Neurol. (2021) 12:628557. doi: 10.3389/fneur.2021.628557, PMID: 34113303 PMC8185273

[15] Zhao G Gu Y Wang Z Chen Y Xia X. The clinical value of inflammation index in predicting ICU mortality of critically ill patients with intracerebral hemorrhage. Front Public Health. (2024) 12:1373585. doi: 10.3389/fpubh.2024.1373585, PMID: 39157528 PMC11327062

[16] Du M Xu L Zhang X Huang X Cao H Qiu F. Association between inflammatory burden index and unfavorable prognosis after endovascular Thrombectomy in acute ischemic stroke. J Inflamm Res. (2023) 16:3009–17. doi: 10.2147/JIR.S419087, PMID: 37489151 PMC10363388

[17] Xie H Ruan G Ge Y Zhang Q Zhang H Lin S. Inflammatory burden as a prognostic biomarker for cancer. Clin Nutr. (2022) 41:1236–43. doi: 10.1016/j.clnu.2022.04.019, PMID: 35504166

[18] Xie H Ruan G Wei L Deng L Zhang Q Ge Y. The inflammatory burden index is a superior systemic inflammation biomarker for the prognosis of non-small cell lung cancer. J Cachexia Sarcopenia Muscle. (2023) 14:869–78. doi: 10.1002/jcsm.13199, PMID: 36852672 PMC10067487

[19] Smith CJ Kishore AK Vail A Chamorro A Garau J Hopkins SJ. Diagnosis of stroke-associated pneumonia: recommendations from the pneumonia in stroke consensus group. Stroke. (2015) 46:2335–40. doi: 10.1161/STROKEAHA.115.009617, PMID: 26111886

[20] Vandenbroucke JP Elm E Altman DG Gøtzsche PC Mulrow CD Pocock SJ. Strengthening the reporting of observational studies in epidemiology (STROBE): explanation and elaboration. Ann Intern Med. (2007) 147:W-163–94. doi: 10.7326/0003-4819-147-8-200710160-00010-w1 17938389

[21] Agnihotri S Czap A Staff I Fortunato G McCullough LD. Peripheral leukocyte counts and outcomes after intracerebral hemorrhage. J Neuroinflammation. (2011) 8:160. doi: 10.1186/1742-2094-8-160, PMID: 22087759 PMC3254078

[22] Di Napoli M Godoy DA Campi V del Valle M Piñero G Mirofsky M. C-reactive protein level measurement improves mortality prediction when added to the spontaneous intracerebral hemorrhage score. Stroke. (2011) 42:1230–6. doi: 10.1161/STROKEAHA.110.604983 21474813

[23] Brunswick AS Hwang BY Appelboom G Hwang RY Piazza MA Connolly ES Jr. Serum biomarkers of spontaneous intracerebral hemorrhage induced secondary brain injury. J Neurol Sci. (2012) 321:1–10. doi: 10.1016/j.jns.2012.06.008, PMID: 22857988

[24] Song Z Lin F Chen Y Li T Li R Lu J. Inflammatory burden index: association between novel systemic inflammatory biomarkers and prognosis as well as in-hospital complications of patients with aneurysmal subarachnoid hemorrhage. J Inflamm Res. (2023) 16:3911–21. doi: 10.2147/JIR.S416295, PMID: 37692059 PMC10488670

[25] Sun Y You S Zhong C Huang Z Hu L Zhang X. Neutrophil to lymphocyte ratio and the hematoma volume and stroke severity in acute intracerebral hemorrhage patients. Am J Emerg Med. (2017) 35:429–33. doi: 10.1016/j.ajem.2016.11.037, PMID: 27876538

[26] Ray S Kumar V Biswas R Ojha VS Bhushan D Kirti R. Neutrophil-to-lymphocyte ratio as a prognostic marker of functional outcome in patients with intracerebral hemorrhage (ICH) and its comparison with ICH score: a hospital-based study. Cureus. (2024) 16:e69350. doi: 10.7759/cureus.69350, PMID: 39398783 PMC11471280

[27] Guo P Zou W. Neutrophil-to-lymphocyte ratio, white blood cell, and C-reactive protein predicts poor outcome and increased mortality in intracerebral hemorrhage patients: a meta-analysis. Front Neurol. (2023) 14:1288377. doi: 10.3389/fneur.2023.1288377 38288330 PMC10824245

[28] Lattanzi S Cagnetti C Provinciali L Silvestrini M. Neutrophil-to-lymphocyte ratio predicts the outcome of acute intracerebral hemorrhage. Stroke. (2016) 47:1654–7. doi: 10.1161/STROKEAHA.116.013627 27165957

[29] Di Napoli M Godoy DA Campi V Masotti L Smith CJ Parry Jones AR. C-reactive protein in intracerebral hemorrhage: time course, tissue localization, and prognosis. Neurology. (2012) 79:690–9. doi: 10.1212/WNL.0b013e318264e3be 22855859

[30] Aronowski J Zhao X. Molecular pathophysiology of cerebral hemorrhage: secondary brain injury. Stroke. (2011) 42:1781–6. doi: 10.1161/STROKEAHA.110.596718, PMID: 21527759 PMC3123894

[31] Woiciechowsky C Asadullah K Nestler D Eberhardt B Platzer C Schöning B. Sympathetic activation triggers systemic interleukin-10 release in immunodepression induced by brain injury. Nat Med. (1998) 4:808–13. doi: 10.1038/nm0798-808, PMID: 9662372

[32] Dirnagl U Klehmet J Braun JS Harms H Meisel C Ziemssen T. Stroke-induced immunodepression: experimental evidence and clinical relevance. Stroke. (2007) 38:770–3. doi: 10.1161/01.STR.0000251441.89665.bc, PMID: 17261736

[33] Kuhlmann CR Librizzi L Closhen D Pflanzner T Lessmann V Pietrzik CU. Mechanisms of C-reactive protein-induced blood-brain barrier disruption. Stroke. (2009) 40:1458–66. doi: 10.1161/STROKEAHA.108.535930, PMID: 19246692

